# Hernia Cerebri

**Published:** 1862-07

**Authors:** R. L. Rea

**Affiliations:** Prof. Anatomy Rush Med. College


### CHICAGO MEDICAL JOURNAL.

VOL. V.] JULY, 1862. [No. 7.

### ORIGINAL COMMUNICATIONS.

### HERNIA CEREBRI.

By R. L. REA, M. D., Prof. Anatomy Rush Med. College.

Few diseases in the entire range of surgical knowledge have received so limited a share of the attention of the profession as Hernia Cerebri. This is particularly true of this country. I became convinced of this in searching for materials to aid in the preparation of the present article. This may be accounted for principally in the rarity of the disease, and yet diseases equally rare and more obscure are not so much neglected. It cannot be from the discouraging fatality of the disease, for in Hydrophobia and Tetanus we have mortality typified, and still these receive a liberal share of attention.

The subject of Hernia Cerebri claims additional attention at the present, from its probable increase in military surgery. For it cannot be that the thousands of sabres and the hundreds of thousands of muskets and other emissaries of death now in use, will fail to do some ragged and unscientific trephining. One so interesting, so little studied or understood, and withal so capable of profitable study, should not be overlooked in the vast field now open before us.

Objections have been urged to the name Hernia Cerebri, from the fact of disbelief in the nature of the tumor.

The term Hernia applies to any tumor formed by the escape of any viscus, or part of the same, from its accustomed cavity. The term Cerebri refers to the Brain. Many doubting the character of the tumor or finding exceptional cases in which it was a fungus growth from the brain, have disallowed its cerebral nature, and denominated it Fungus Cerebri.

In injuries of the cranium where a tumor forms, in a vast majority of cases—in fact the rule is, that it is the substance of the brain—we would see in that a sufficient reason for retaining its name. There are rare cases in which none of the cerebral substance may be embraced in the tumor, but that should no more change the name of the disease than the exceptional absence of membrane should change the name of Membranous Croup, or the name Hysteria because of its occasional occurence in men.

This form of disease can occur under but one condition, the want of cranial support for the brain—an absence of the walls of the skull and the membranes enclosing it.

This may occur through congenital deficiency of the parts, or by their accidental destruction, either by force applied, or syphilitic caries, or it may follow the application of the trephine.

When coming from either of these causes, the membranes are defective primarily, or are destroyed, giving way before the hernia takes place.

These, then, are the circumstances under which Hernia of the Brain must happen, but it by no means follows that under the same apparent conditions, hernia will always occur.

Here we meet with one of the most perplexing facts in connection with this fertile and seemingly contradictory affection. In one instance we have an injury to the skull requiring a removal of a portion of it—the membranes are found to be lacerated, and the operation is soon followed by a protrusion of the brain through the newly made opening. Another instance is observed where, so far as we are able to see, precisely the same conditions are present, skull removed, membranes torn, and equal violence applied; and yet no symptoms of Hernia present themselves.

What are the circumstances involving this difference of result?

In order to determine this, we must first determine, if we can, the cause of the protrusion, and before doing this it will be indispensable that we know the nature of the protruded substance.

Reversing the order of the inquiries as stated, then we will first inquire into the substance which we see appearing through the opening. The inquiry comes in properly here, for if it be a true fungus, no force will be needed to cause its increase in size beyond ordinary growth; but if it be brain, another agency must be present.

It might interest us much and profit us little to discuss the opinions held by different observers as to its nature. Suffice it to say, some have maintained it to be granulations of a fungus character, forming on the surface of the brain; others that it was semi-organized blood, while by others it is decided to be brain substance itself. While the latter opinion is now almost universally conceded to be the true one, we must not too hastily condemn the seeming want of care in the observations of those who hold the former. The tumor seen after it is fully formed, is well calculated to deceive any one relying upon its external appearance. From the color of brain matter when it first appears, it speedily becomes covered with effused blood, which darkens by exposure, so that the tumor soon has the color of a clot of blood. When ulceration begins, and this effused blood is thrown off, it assumes the appearance of an ordinary ulcer.

The Microscope, however, is the true test. Whatever we might decide from appearances, when the substance is carefully examined in this way, and decided to be of any given kind, it sets inquiry at rest.

Examinations made with the microscope unmistakably show that the matter which appears through an injury done to the cranium, is in almost every case true brain substance. And yet while this is true, they quite as unmistakably show that in rare cases no brain matter whatever is detectable, the mass being composed of fungus generations or partially organized blood.

Some cases occur in which it is a combination of either or all these elements. This has been most satisfactorily confirmed by the observations of Dr. Adam Clark and Dr. C. E. Isaacs, of New York.

Before seeing an account of the examinations of these gentlemen, I had my mind very fully made up that it could be nothing else than brain, coming to my conclusion from that very satisfactory kind of evidence—my own senses. However easily we are deceived, we are very apt to place implicit reliance in any thing as seen by ourselves, and strong proof is required to disabuse us of our errors. Two cases which had come under my own observation, furnished me with the most conclusive proof that it could be nothing else than brain.

In one of these, the one appended to this article, I witnessed immediately on the removal of the skull, which had lacerated the membranes, the commencement of the tumor. The substance of the brain was easily recognizable, without the possibility of a mistake in its consistence and color—and within the hour or so that I remained with the case, it was plainly to be seen slowly increasing with each arterial pulsation. In the other case referred to, I made an opening through the Dura Mater for the escape of blood. As soon as the contained blood had escaped, the brain substance began to make its appearance through the opening—the patient dying however, in six hours, before a tumor of any size had developed itself.

In the former case, in which I removed a portion of the tumor, the colors of the white and gray matter were clearly distinguishable.

If any thing further were needed to confirm an already proven fact, it might be mentioned that in cases where portions of the tumor have been removed, and death following, a depression will be found corresponding with the removed part. While this furnishes evidence of its brain nature, it also furnishes the objection that the amount of depression found seldom bears any just proportion to the amount of tumor removed. This is accounted for when we remember that the brain is intensely congested, and that the part which is extruded is greatly hypertrophied, and a part which was large on its removal, containing more blood and less than its normal amount of brain, leaves a proportionately small depression on the surface of the brain, when death taking place, and congestion removed, they occupy their normal limits.

Having settled pretty conclusively the nature of this tumor, we will now direct our attention to the more fertile inquiry concerning the agency or agencies at work which produce it.

Suppose a portion of the cranial walls with their soft parts destroyed, from any cause, what constitutes the active agency in causing the brain to protrude through the opening thus supposed to exist? Various as were the theories with regard to the nature of the material of the tumor, they are limited, compared with conjectures as to its protruding force... Of one thing we can have no doubt, that there is an active force somewhere, tending constantly to dislodge the brain. It is not a growth in but the fewest cases, as we have demonstrated. Neither does the brain grow out of or inherently tend to leave its cavity, for every natural law tends to keep it there. Neither can it be that the brain possesses a vernacular ability to extricate itself or pour out as fluids. Every thing tends to show that there is an agency independent of but acting on the brain.

Most of the opinions held have reference to inflammatory action going on in the brain. All, in fact, either have reference to inflammation or its products. Some of them are embraced in the following extract taken from Dr. Weir's article in the New York *Journal of Medicine* for November, 1859:

"Stanley says that to produce a Hernia Cerebri, there must be an increase in volume of the contused parts, caused either by a general distension of the blood vessels of the brain, or by the addition of some new matter, as water or pus.

"Samuel Cooper states that Hernia Cerebri is a disease connected with deep seated changes throughout a great part of the brain, conjoined with removal of bone. The changes alluded to may be supposed to cause an increase in the general contents of the skull, and thus a disposition to protrusion as rapid as the serum and other fluids are effused.

"Brodie, in compound fractures of the skull, the brain having lost the support which it derives from the dura mater, and having its vessels loaded with blood, would probably protrude in the form of Hernia Cerebri.

"Guthrie, it arises from a low grade of inflammation of the brain.

"Miller, with Drs. Laurie and King, ascribe its appearance to disorganization of the brain by inflammation.

"Sedillot says the appearance of the Hernia is caused by a distended ventricle.

"Propulsion from the arteries is adopted by Flourens and Dr. N. R. Smith."

Thus it will be seen that inflammation, its preludes and accidents, are foremost in the minds of the writers mentioned, as the cause.

Guthrie pronounces it a "low grade of inflammation," with what propriety I am unable to determine. Its occurrence in the robust, and during the continuance of all the initial and most violent symptoms, would seem to disprove this, as well as the fact of its rapid formation after the injury, as most frequently takes place.

The ideas advanced by Drs. Miller, Laurie and King would seem to be disproved by the fact of its immediate occurrence in some cases without time necessary for "inflammatory disorganization."

It is difficult to understand what the exact meaning of

Cooper's "deep seated changes" is, unless it is inflammation and its products.

The "distended ventricle" of Sedillot, is of course from serum or pus, consequent on inflammation.

Stanley has the merit of at least being clearer if not nearer right, though he does not mention inflammation as such.

The succession of phenomena, as they would naturally take place in this accident, seem to me to be as follows: An individual receives a blow on some portion of the head of sufficient force to drive in the skull and lacerate the membranes. The patient falls insensible, and if the injury be not too severe, reaction will come on, and he will return to consciousness—or compression may come on preventing this. What changes go forward in the brain? At the first the stun or shock causing insensibility, during which time the circulation will be depressed, followed in a longer or shorter time by reaction with its increased circulation, and afterward the ordinary succession of inflammatory phenomena. Within a few hours, at most, the patient will be seen, the skull trephined, upon the removal of the bone a tumor will soon occupy the opening, which may be seen to gradually increase in size, with each pulsation of the arteries a distinct swell will be perceived, which will as regularly subside with the cessation of the arterial action. This was most distinctly marked in the two cases which have come under my notice.

First, then, let us determine, if we can, what are the causes at work to produce this calamity, and afterwards decide as far as possible as to the difference in the causes, or their amount, which in one case will produce a Hernia and in another are followed by no such evil effect.

The cranial cavity is a vacuum plenum. There may and I think is, a variation often as to the relative amounts of the contents of the cranium. The quantity of fluids contained in that cavity varies at different times, under different circumstances. I mean that more fluid can be condensed into the cranial cavity than is usually present or necessary for its normal and healthy action, and this may take place within certain limits without permanent detriment. This fact, though formerly much mooted, seems now well settled. We are of course justified in assuming that the quantity of brain, or the size of its case, does not vary.

We are all familiar with the well known effect of exhausting diseases on children, as shown by its effect on the fontanelles. In the well fed, hale infant, we have at the situation of the anterior fontanelle, the plump, protruding, pulsating brain, arising on a level with or even beyond its bony surroundings at each succeeding arterial pulsation. Let an exhausting disease of any kind seize our little subject, and speedily as the progress of the disease marks itself will our cerebral barometer sink. The brain, unsupported by bone at this point, takes recess from its accustomed compression by extruding with each action of the heart. This will of course depend on having a natural condition of the cranial contents, and then adding the injecting force of the heart upon the blood thrown into the brain at each beat. The combined dilation of the arteries acting on the brain will produce the phenomena we have noticed. Add to the ordinary cranial contents serum or other material, and of course you increase the protrusion to its limits, or compress the brain proportionately. Diminish the amount of complemental serum surrounding the brain, which is sometimes termed the "Hydrostatic bed," or weaken the action of the heart, and no such effect is observable, for the sum of the dilatability of the arteries will only distend the cranial contents to their ordinary level, and instead of a protruding of the brain through the fontanelle, it will only rise to its medium level or fall below it, and exhibit its characteristic appearance under depressing diseases. It is most evident that this peculiar action of the bran in infants is owing to no vessel immediately in the vicinity of the fontanelle, for there is no artery upon whose dilation it could depend, and the action being synchronous with the heart, must depend on it. That the brain sustains vascular pressure constantly, is well proven by the fact of its sudden removal causing swooning and a cessation of its function. The effect of any abnormal condition causing an increased flow of blood to the brain, or increasing the force of the heart, must add to this pressure. In the sleeping and waking states, a difference is noticeable in cases where absence of a portion of the skull has allowed the observation, in the sleeping state subsiding below the level, while upon waking the mental stimulus causing an increased flow of blood, fills up the organ to its usual wake level.

From these facts, it is fair to conclude that a force acting upon the brain as expansion of the arteries is capable of comcompressing the brain to a certain limited extent, and under certain circumstances causing it to protrude from its cavity. The brain, accustomed to receive the shock of the heart's action through the arteries, is supported by the membranes and osseous walls; and when a portion of the brain is deprived of this support, the tendency would of course be to give way before the pressure. It is with the consistence of the brain in mind that we could compare this action to that of the piston of a syringe in expelling its contents. A quantity could be expelled by the introduction of the piston, the size of the piston governing the amount thrown out. However small the piston, this would be true, and carrying out the comparison, the force requisite to expel would depend on the consistency of the substance to be expelled.

Hernia of the Brain, as a rule, follows the application of violence to the head. An amount of violence necessary to fracture the skull will seldom if ever fail to produce inflammation, with its attendant congestion and hyperemia. These effects will cause an increased amount of blood to be sent to the brain, and with additional force, which is but a repetition of the old maxim, "Ubi irritationis, ibi fluxus."

I think we are warranted in concluding that the force of arterial action being sufficient to drive the brain before it in a healthy condition, is abundantly able in a diseased condition of it, when we add the increased action of the heart, and the exaggerated amount of blood which determines to it, and still further if we diminish its capability of compression by condensing it by inflammatory or congestion effusions. The idea I attempt to convey is this: The dilating arteries find a recess for their dilation, so to speak, in the compressibility of the brain. Should this be prevented by an effusion of serum, for example, previously acting on the brain, their expansion would be correspondingly limited, and the brain of a doughy consistence impressed upon at one point will extend itself naturally in that direction in which there is no resistence.

Hence I conclude that the protrusion of brain in this accident is caused by the force of the blood driven into it, the effect of the injury inflicted, which may or may not be accompanied by inflammation, and aided by its effusions.

This being conceded, we now take up that other question previously alluded to, Why in injuries involving a removal of a part of the skull, Hernia Cerebri does not invariably take place?

My friend, Dr. Bogue, of this city, was called to a boy fourteen years of age who had been kicked by a horse, knocked down, comatose, in which he found a considerable portion of the skull in the right parietal region comminuted and depressed, which he removed to find the membranes lacerated and portions of brain forced through their opening. Within twenty hours he was conscious, and from that he had not an untoward symptom. He made a rapid, safe, and perfect recovery.

Here is the counterpart of my own case to all appearances, and yet Hernia did not take place.

As to whether a Hernia will form in any given case, it appears to me will depend on one or more of the following conditions:

First, the amount of original injury. The liability of the brain to be forced through any given opening will of course depend upon the degree of its solidity or consistence. To resume our illustration of the syringe, the thickness or thinness of its contents will determine the force necessary to expel them. We can easily see the difference in trying to throw through the syringe successively water, syrup, and tar, the first passing through with perfect ease, the second requiring more force, and the latter scarcely capable of going through at all, though all are liquid. So I take it will the ability of the forces at work in extending the brain depend on the solidity of the brain substance.

The amount of force occurring in the production of injuries of this kind will of course vary very much. In some being simply enough to fracture the bones, without being powerful enough to extend its destructive effects to the substance of the brain in the injury of its structure. In others, as falls from heights, violent blows, &c., the injury not only breaks the bones and lacerates the membranes, but extends its influence to the brain, producing contusion of its substance, and if examined will be found soft and pulpy. The examination of one of my cases proved the existence of this condition; the brain corresponding with the seat of injury being completely broken down and in a softened and semi-fluid condition.

It is evident then that this softened state in immediate juxtaposition with the opening will greatly predispose to its occurrence. In addition to the condition of the brain thus produced by the original force, the other conditions accompanying violent injuries will be present—as a high grade of congestion, inflammation, &c., which we have concluded are the active agents at work in the production of the Hernia, and therefore will give as additional reason to expect the occurrence of this accident, the brain being supposed to swell when unrestrained under inflammation as other organs.

Second, consistence of the Brain substance as depending on abnormal softness or age.

Any one accustomed to making post mortem examinations must have noticed the different degrees of hardness in different brains. Some are of a firm and comparatively solid consistence, while others are but little harder than thick cream. This, with other conditions, give us our presumed predisposition.

An idea with reference to the enlargement of the skull and the connection of age with Hernia is advanced by Humphrey in his work on the Skeleton.

The head of the child as we know is disproportionately large compared with the other parts of the body. The economy of this arrangement he contends is that from the conformation of the cranium its enlargement must take place very slowly owing to the fact that it can increase in size only by the growth which takes place on the contiguous margins of the bones, and by the resorption from their inner and deposition on their outer surfaces.

The enlargement of the capacity of cranium then, must needs be very slow. According to his theory, the head of the child, large in the first place, contains a brain with a large per cent. of water added to the normal elements of brain, and as a consequence is soft and flaccid. Instead of the brain and head enlarging rapidly as the developing mental faculties demand new and increasing supplies of brain substance, instead, I say, of the brain's enlarging to supply this entire demand, while it can grow but slowly, it adds to its power by condensing its structure, so that were it not to enlarge at all it might contain a considerable addition of cerebral matter.

The Hernia Cerebral part of this fact is that the brain of a child is softer than the adult, and that it continues so until the complete development of the other parts of the body. Youth then, I infer, is a predisposing cause of the disease. My idea is borne out by the facts in the cases collected by Dr. Weir, before alluded to. Of fifty-five cases, the average was twenty years. When we consider that those very young are seldom exposed to dangers which would likely produce this injury, that they occur from severe blows received in fights, falls from heights, and in mechanical pursuits not followed by children, we can understand this seeming high average age. In Dr. Buck's cases, seventeen were under twelve and twenty-six under twenty years of age. Ericksen in his work mentions the fact of its more frequent occurrence in children.

Still another circumstance governing its occurrence will be the size of the original osseous opening. As the enlargement of the opening may be regarded as a part of suggested treatment, the philosophy of it will more properly come in there, to which place the reader is referred.

The occurrence of Hernia, after the application of the Trephine, requires some notice before leaving the productive causes.

Here it might be said we lack the inflammatory action necessary to produce this effect. We must remember that the Trephine is always used to remedy a disease of some kind effecting the brain, and applied in its immediate vicinity. What condition the brain may be in in cases in which it occurs cannot of course be determined, but I think we may conclude that one of the conditions mentioned, either a distending force applied to the brain or an unnatural flaccidity of it. The brain under the influence of disease may have softened, and the application of the trephine would be followed by more or less vacular excitement.

Dr. Gross, in his work, mentions that the worse case he witnessed followed syphilitic destruction of the walls and membranes. He does not state the nature of the tumor, however, whether it was brain matter or fungus granulations. If the former, the same explanation would naturally follow for this case as the last—softening and vascular action.

The question would very properly be asked here, Why it is that Hernia occurs often a number of days after the receipt of the injury? Why does it not take place immediately after the injury is inflicted? This class of cases I think can be accounted for, not from the violent primary congestive action or from contused condition of the brain, but that it will be found to happen in connection with the products of inflammation, either in the form of an abscess or serous effusion, as we have in a distended ventricle, or a collection of blood. Dr. Buck says, "in all the cases he examined an abscess was found in the substance of the brain or upon its surface in the immediate vicinity of the Hernia." The formation of these depots of serum and pus would correspond in point of time with the date of the delayed Hernial protrusion, and I think in justice might be assigned as the cause, when the violence of the vascular action does not produce it at an earlier period.

The prognisis in this affection is very uncertain. With seemingly small injuries, many die, and those apparently helplessly hurt, recover. This depends, I doubt not, on the difference between the apparent and real amount of injury. In individuals where there is not too much inflammatory element, where the injury is not so severe as to give rise to extensive constitutional disturbance and the constitution good, we have a right to anticipate a favorable result with prompt and efficient treatment. Too often the amount of injury received by the brain is beyond our cognizance and from a vitiated constitution thwarting our efforts, the result is fatal. I could by no means agree with Dr. Gross in the opinion, however, that "recovery is an extremely rare occurrence in any case, however simple," when I remember that sixteen out of the thirty-three cases of Dr. Buck recovered.

The length of time occupied by this malady is subject to much variation, the patient dying occasionally as early as the first day, and again lasting for months, dying worn out of nervous irritability; in the former case the the result of the overwhelming nature of the injury, and in the latter of centric irritation.

John McNulty, Irish boy, aged 14, was engaged driving a horse hoisting coal from a vessel; while thus occupied, he got into a quarrel with another boy, who seizing a piece of coal threw it at him, striking him a little above and outside the left frontal eminence. He was taken up and carried home insensible, and Dr. Baltzell called to see him nextday, October 4, 1859. I accompanied the Dr. to see him and found him only partially conscious, pulse 128, skin hot, pupils dilated, with an incision in the locality named. Upon examination, we found a fracture, and the fragment depressed. By request of

Dr. B., I removed the portion of bone, which was readily done by enlarging the incisions, the fragment being entirely detached was easily removed with the bone forceps. It was irregularly triangular, the sides of which were as near as may be one and a half inches. Upon removing the bone, the membranes were found cut through to the extent of half an inch in length. The wound bled but little and was closed in its upper portion. Before closing the wound, I noticed in the situation of the opening through the Dura Mater, what I supposed to be a portion of brain beginning to protrude. It was of a greyish white color, and pulsated with the action of the heart. It being my first experience with this class of affection, I did not feel sufficiently certain of its character to decide on its treatment. Accordingly we contented ourselves with closing the wound, as I have said, applied cold water to the head, and ordered a saline cathartic. We visited him about 5 P. M. same day, and found him about same, except consciousness, which seemed improved, though the cathartic had not operated. The tumor which we noticed at our last visit, is increasing in size. Cathartic ordered repeated, and cold continued. The next morning, (5th Oct.,) upon visiting him, we found there had been a free action of the cathartic, with a decided improvement in his general symptoms. He was entirely conscious, pupils more natural, skin cooler, and converses sensibly. Complains of pain in his head, not particularly located at the wound. The Hernia has increased in size until it now amounts to that of a small hazel nut. The Dr. having kindly given the surgical management of the case to me, I concluded to shave off the protruding portion of brain, which I did on a level with the surrounding part, upon doing which I noticed an enlargement of the original opening through the Dura Mater. I then prepared a compress the size and shape of the osseous opening, saturated it with Mur. Tr. Iron and applied, it, covering it with another dry one, both of which were firmly bandaged on. The removal of the brain did not cause the slightest pain, nor was it followed by any hemorrhage.

The next day, 6th, we found him doing well, no inconvenience from the dressing, had suffered no additional pain, slept moderately well, still somewhat feverish, but every way seemed quite as well as we could expect. Upon removing the dressing, we found it had retained the brain in position perfectly, and had fulfilled my expectations in the formation of a crust over the surface of the brain. Dressing applied as before.

On visiting him the 7th, he seemed much improved bright, and cheerful, no pain or uncomfortable feeling about his head; his wound appeared in unexpectedly good condition, the brain being quite to its level, pulsation in it almost ceased, with so many good symptoms and the brain so firm, I was misled by appearances, removed my dressing, closed the wound over it, and applied cold water dressing. A visit that evening proved I was mistaken in the firmness, for unrestrained it had already began to enjoy its liberty, and was bulging. The following morning found it protruding beyond its bony margins, the Dura Mater having given way probably under the influence of the Tinc. Iron, the tumor completely filled the opening, being in substance the size of a medium hickory nut. This I removed completely as before with no different results. No inconvenience was experienced by the patient, either at the time of the excision or afterward. The Tr. Iron was reapplied and continued from day to day by Dr. Baltzell, with whom I saw the case frequently until the cure was perfect. Within a few days after the application of the Tr. Iron, suppuration began on the surface of the brain, the crust which was formed loosened, but it was allowed to remain, and a fresh compress saturated with the Iron applied; the crust thus formed being an exact mould of the surface of the brain, gave equal pressure on all parts of it through the compress applied over it. Upon the removal of the brain each time, there was no diffiulty in distinguishing it, the color of the white and gray matter being easily recognized.

The case progressed favorably, though it was several months before he entirely recovered, and I think nearly five months before the compress was left off. The experiment of leaving it off was never repeated until the brain became firm.

He recovered perfectly, no paralysis or unpleasant symptom remaining, the opening through the skull closing by fibrous membranes, and that it has left no mental defect is proven by the fact that he now pursues the calling of a newsboy in our streets, and is one of the smartest of that crafty class. In the application of pressure no unpleasant symptoms manifested themselves, though it was firm and constant. I saw the boy a few days since, and find the opening closed by a very thin layer of tissue, and can easily be compressed. No appearance of any thing firmer being formed, though it is near three years since the accident.

On no part of our subject is there such direct antagonism of opinion as on the treatment, our most distinguished authorities taking directly opposite views. This difference is mainly on the questions of excision and compression.

Guthrie disapproves excision, and depends on moderate pressure, lightly applied while the tumor is increasing, augmented while it is decreasing.

Cooper and Miller advocate same.

Stanly says compression is out of the question, and is undecided as to the use of the knife.

Ericksen advocates slicing off the brain to the surrounding level, and applies firm and sustaining compression.

From having treated my case successfully, I naturally sustain the views of the latter. There are many cases in which a modification of the treatment would be rendered necessary. As, for instance, in which a tumor had already formed of unusual dimensions, as occurred in the case reported by Dr. Mears, of Indianapolis, in the Northwest Medical Journal for October, 1848, when it arrived at the unusual size of of "a section of a middling sized orange." In this case no other treatment is reported except slicing small portions away from the surface of the tumor, the patient recovering, slightly paralytic, on the opposite side. Removing so large a portion of brain could be nothing but deleterious. In such cases, with moderate slices could be well combined styptics, as the Tinc. Iron, and moderate and judiciously applied and sustaining compression. When, however, the surgeon is called at the time of the accident, there is but little necessity for the formation of such tumors. If the proper remedies are used, I should have little fear of any such result. As soon as the protrusion is discovered, prompt steps should be taken for its arrest. Antiphlogistic measures should be adopted and carried as far as the case admits, and the support of a compress applied over the part. The kind of a compress there is a variety of opinions, but I think a linen compress as good as any thing firmer, being entirely sufficient to retain the brain, and can be saturated with any desirable fluid. I used the Mur. Tr. Iron on account of its astringent qualities, and it answered so perfectly I should feel much confidence in repeating it.

There is a remedy which has since occurred to me in studying the cause of this disease, that I think cannot fail to exercise a controlling influence over it. I refer to Veratrum Viride.

If, as I have supposed, most of the curable cases are caused by arterial action, a ligation of the forcing organ cannot fail to exercise the most happy influence, and I should feel great confidence in this as one of my remedies in another case.

The application of compression to a tumor of any size previous to excision, I deem of doubtful utility. I think the suggestion of Dr. Buck eminently true. "May not," says he, "this protrusion be a salutary resource of nature to relieve the brain (often only temporarily) from the compression which an increased afflux of blood might occasion;" and therefore it could be nothing but the worst of practice to force back into the skull that which nature, under desperate circumstances, was compelled to cast away. After the brain was once extruded, and the vessels had expanded under the relaxation, the risk of compression of the brain would be vastly greater in repositing the brain than under the original support of a compress.

As a final addition to our treatment, I may mention the suggestion of some, that an increase of size of the original opening would suffice to prevent further increase of size of the hernial mass. This idea is based on the supposition that were the compressed brain allowed to expand itself sufficiently by the removal, or enlargement of its walls to the required extent, it would of course have no tendency to form a Hernia, because it would then expand symmetrically. So we reason if any considerable portion of skull were removed, a corresponding portion of brain could expand and would afford room for the expansion of the distended vessels, and render its occurrence correspondingly improbable. Suppose, for instance, the brain had no restraining case of any description, and could expand and diminish in size with each varying circumstance of the circulation, then of course no Hernia could occur. If one half the brain case were removed neither would it, because there would still be room for adequate expansion. May not this have been the reason that in Dr. Bogue's case there was no Hernia, the opening being of unusually large size? I have measured the bones and find not less than 2½ square inches removed. This treatment embraces the idea that a portion sufficient to give room for this enlargement would avoid the increase in size altogether. How far this has been attempted, I am unable to say; but it appears to me that in order to give the needed room, the Dura Mater must needs be removed to a corresponding extent, for we know Hernia does not form unless it be broken, owing to its want of elasticity. No doubt the principle is correct, but with this fact in view, it looks to me to be hazardous practice.

The exposure of a portion of brain of requisite size, for such a purpose, with the possible complications to which it may give rise, and the encouragement given to prompt treatment, would lead me to doubt the expediency of a practice which would add a risk to an already very mortal accident.

